# Effects of Step Length and Stride Variation During Forward Lunges on Lower-extremity Muscle Activity

**DOI:** 10.3390/jfmk10010042

**Published:** 2025-01-22

**Authors:** Rafael F. Escamilla, Irwin S. Thompson, Robert Asuncion, Jacqueline Bravo, Tiffany Chang, Taylor Fournier, Hannah Garcia, Emily Hockenbery, Kyle Nagasawa, Joan Ozor, Hannah Snoeberger, Kevin E. Wilk, Mario Bizzini

**Affiliations:** 1 Department of Physical Therapy, California State University, Sacramento, CA 95819, USA; irwin.thompson@csus.edu (I.S.T.); robert.asuncion@csus.edu (R.A.); jmcorona@csus.edu (J.B.); tchang2@csus.edu (T.C.); tfournier@csus.edu (T.F.); hannahgarcia@csus.edu (H.G.); emilyhockenbery@csus.edu (E.H.); kcnagasawa@csus.edu (K.N.); joanozor@csus.edu (J.O.); hsnoeberger@csus.edu (H.S.); 2 Champion Sports Medicine, Birmingham, AL 35243, USA; kwilkpt@hotmail.com; 3 Human Performance Lab, Schulthess Clinic, 8008 Zürich, Switzerland; mario.bizzini@kws.ch

**Keywords:** electromyography, lunging, muscle recruitment

## Abstract

Background: The forward lunge is a closed-chain weight-bearing multi-joint exercise simulating the activities of daily living, such as walking or stair climbing, which mainly activates hip, knee, and ankle musculature and is also used by athletes and other individuals to train lower-extremity musculature. Objectives: The purpose of this study is to compare lower-extremity muscle recruitment patterns between stride and step length variations in forward lunges. Methods: Twenty participants had a mean (±SD) age, mass, and height of 26 ± 6 y, 79 ± 8 kg, and 176 ± 7 cm, respectively, for males, and 27 ± 4 y, 62 ± 6 kg, and 161 ± 7 cm, respectively, for females. All participants used their 12-repetition maximum weight while performing a short step and long step forward lunge with a stride (striding forward and pushing back to the starting position) and without a stride (lunging up and down with feet stationary). During each lunge variation, surface electromyography (EMG) data were collected from the quadriceps, hamstrings, gastrocnemius, hip adductors, gluteus maximus, and gluteus medius muscles, and then normalized as a percent of each muscle’s maximum voluntary isometric contraction. A repeated measures two-way analysis of variance was employed (*p* < 0.01), with step length and stride comprising the two factors. Results: The following had no significant interactions: (1) quadriceps, hamstrings, gastrocnemius, hip adductor, and gluteus maximus EMG activities were significantly greater in lunges with a long step compared to lunges with a short step; and (2) gluteus maximus and gluteus medius EMG activities were significantly greater in lunges with a stride compared to lunges without a stride. The following had significant interactions: (1) gluteus medius EMG activities were significantly greater in lunges with a long step with and without a stride compared to lunges with a short step with and without a stride; (2) quadriceps EMG activities were generally significantly greater in lunges with long and short steps with a stride compared to lunges with long and short steps without a stride, in lunges with a long step with a stride compared to lunges with a short step with a stride, and in lunges with a short step without a stride compared to lunges with a long step without a stride; (3) hamstring and hip adductor EMG activities were significantly greater in lunges with a long step with a stride compared to lunges with a long step without a stride, and in lunges with a long step with and without a stride compared to lunges with a short step with and without a stride; and (4) gastrocnemius EMG activities were significantly greater in lunges with a long step with and without a stride compared to lunges with a short step with and without a stride. Conclusions: Lower-extremity muscle activity is generally greater in forward lunges with a long step compared to a short step, and greater in lunges with a stride compared to without a stride. During the externally loaded forward lunge, high to very high muscle activity occurs in the quadriceps, gluteus maximus, and gluteus medius, thus enhancing muscle hypertrophy and strength in these muscles, while moderate muscle activity occurs in the hamstrings, gastrocnemius, and adductor longus.

## 1. Introduction

Exercise-related movements from daily activities and sports are of major interest for physical therapists, sports therapists, sports researchers, and strength and conditioning specialists. The forward lunge is a closed-chain weight-bearing multi-joint exercise simulating the activities of daily living, such as walking or stair climbing. The forward lunge mainly activates hip, knee, and ankle musculature, and is also used by athletes and other individuals to train lower-extremity musculature [1,2,3,4]. As a strengthening exercise, the forward lunge is one of the more commonly used exercises for sports training, with the goal to improve functional and sport performance [1,5]. Forward lunges are also employed for rehabilitation [6], such as in patients with knee injuries and pathologies, including patellofemoral pain syndrome (PFPS) [7,8,9,10,11], and after anterior cruciate ligament (ACL) injury or reconstruction [3]. The forward lunge can also be used as a part of an injury prevention program to reduce the risk of ACL injuries in athletes [12]. The forward lunge is not only recommended for rehabilitation purposes in young athletes, but also for fitness purposes. It is also beneficial for older adults because it is relatively safe, can be performed at home without supervision, requires minimal equipment, permits unilateral loading, and can enhance quality of life [5,13].

Forward lunges can be executed with various techniques. Common variations in the lunge exercise include different movement directions [1,13,14], long and short step lengths [2,15], varying trunk positions [16,17,18], external loads [2,19,20,21], or unstable surfaces [6,18,22,23,24,25,26]. A long-step lunge often results in the front knee positioned over the front foot at the lowest position of the lunge, while a short-step lunge often results in the front knee moving forward 8–10 cm beyond the toes when the lunge is at its lowest position [15]. These step length variations can be employed as an intensity or loading progression during a rehabilitation or training program. In the traditional version of the forward lunge [3,14,17,23,25], participants step forward with one limb and then lower the body as much as possible, slightly contacting the ground with their rear knee. Once the lowest position is reached, an immediately push backward with the lead limb returns the individual to the standing starting position [3,15]. This is referred to as lunging with stride [15]. Conversely, lunging without stride involves fully flexing and then extending the knees to the lowest and highest lunge positions while the feet remain stationary throughout [15]. Both when athletes or other individuals are first learning how to perform the forward lunge, or during earlier phases of rehabilitation when the objective is to minimize lower-extremity muscle activity or joint forces, clinicians initially introduce forward lunging without a stride, and then over time progress to lunging with a stride [15].

Surface electromyography (EMG) is the most common technique to evaluate the activity of the muscle groups involved in an exercise [14]. From the EMG signal, the timing and magnitude of muscle activity obtained in different conditions can be compared, which provide indications about exercise intensity and workload [14]. Based on this information, the best exercise selection (and associated progressions) may be determined according to training or rehabilitation objectives [14]. Although lower-extremity muscle activity patterns have been assessed in forward lunge studies with various techniques [1,16,17,18,19,22,24], there are no known studies that have specifically compared forward lunges with step length (short versus long step) and stride (with a stride and without a stride) variations. Therefore, the aim of this study was to compare lower-extremity muscle EMG activity as a function of step length and stride variations. The hypotheses were that overall lower-extremity muscle EMG activity would be greater when performing lunges with a long step compared to a short step, and that it would also be greater when performing lunges with a stride compared to without a stride.

## 2. Materials and Methods

### 2.1. Participants

Twenty healthy individuals (ten males and ten females) with no history of knee pathology participated in this study. Participants were recruited by bulletin board announcements, posters, flyers, brochures, and e-mail distributions within the California State University, Sacramento community. Participants had a mean (±SD) age, mass, and height of 26 ± 6 y, 79 ± 8 kg, and 176 ± 7 cm, respectively, for males, and 27 ± 4 y, 62 ± 6 kg, and 161 ± 7 cm, respectively, for females.

Inclusion criteria included each participant being able to perform all forward lunge movements with proper form and technique (as judged by a licensed physical therapist with 30+ years of performing forward lunges) for 12 consecutive repetitions while employing each participant’s 12 repetition maximum (RM) weight. Exclusion criteria was any participant who did not have at least 1 year experience performing the forward lunge exercise with dumbbells, unable to perform the forward lunge pain-free, and any previous history of knee pathology.

To improve the EMG signal quality, all participants had below average or average body fat [27], and was measured by baseline skinfold calipers (Model-68900, Country Technology, Inc., Gays Mill, WI, USA) with suitable regression equations. Mean (±SD) body fat was 13 ± 4% for male participants and 19 ± 3% for female participants. Written informed consent was provided in according to the Institutional Review Board at California State University, Sacramento.

### 2.2. Pre-Test Session and Exercise Description

Each participant attended a pre-test session 1 week before testing, and experimental procedures were reviewed. Each participant’s 12 RM was determined (dumbbells were available between 2.27 kg and 45.45 kg, with increments of 1.14 kg) while performing the forward lunge with a stride using a step length halfway between the forward-lunge-long and forward-lunge-short, and this weight was used while performing the lunge variations during data collection. Given that all participants regularly performed the forward lunge with dumbbells, they all had an idea what their 12 RM would be. All participants were able to determine their 12 RM within 1–3 trials, and were given a 5 min rest interval between 12 RM trials. The 12 RM mean (±SD) dumbbell mass (from two dumbbells, one in each hand) were 46 ± 10 kg for males and 30 ± 7 kg for females.

**Figure 1:**
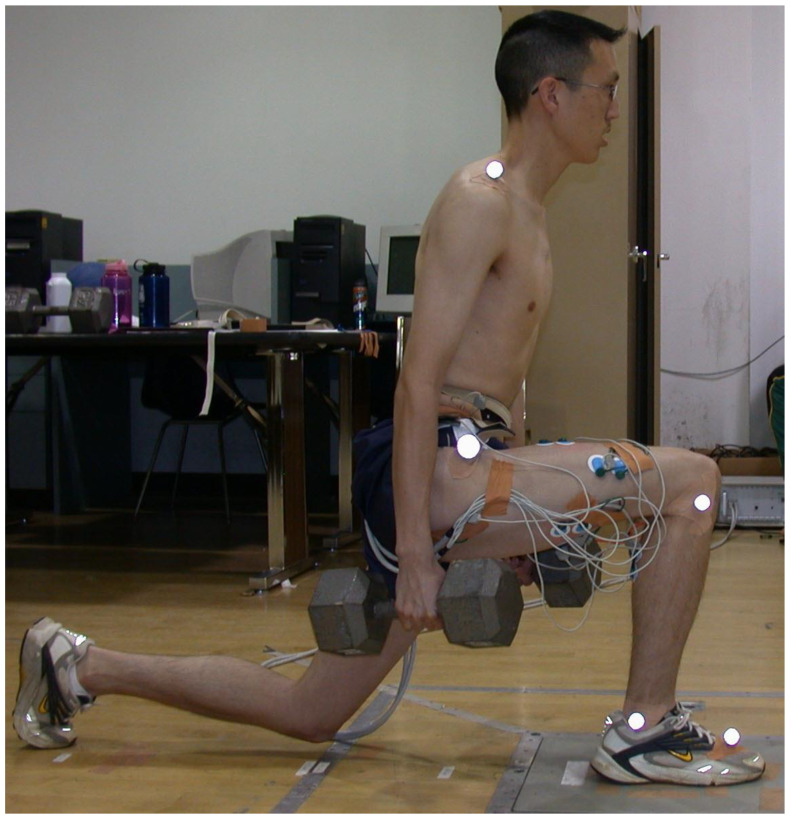
Forward lunge with a long step (forward-lunge-long).

**Figure 2:**
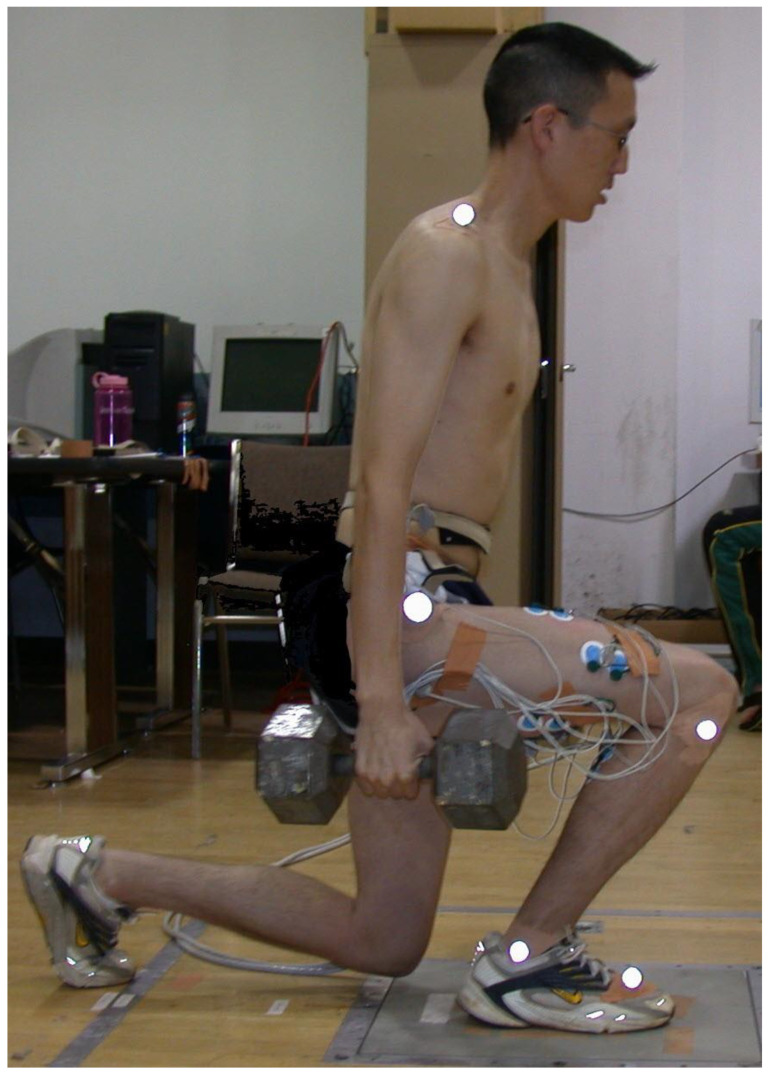
Forward lunge with a short step (forward-lunge-short).

The participants practiced both with stride and without stride forward lunge variations using both a long step forward lunge (forward-lunge-long; Figure 1) and a short step forward lunge (forward-lunge-short; Figure 2). During the forward-lunge-long, each participant employed a step length that caused the right leg (tibia) to maintain an approximate vertical position at the lunge lowest position (Figure 1), thereby keeping the knee over the foot. The mean (±SD) step length (which was measured from the left toe to the right heel) for the forward-lunge-long was 86 ± 3 cm for males and 77 ± 5 cm for females. The forward-lunge-short step length was half of the distance used for the forward-lunge-long, and the shorter step resulted in the anterior surface of the right knee translating forward approximately 8–10 cm beyond the toes of the right foot (Figure 2). While performing the forward-lunge-long and forward-lunge-short, both with and without a stride, maximum trunk tilt forward (which happened near maximum flexion of the front knee) was not controlled, but remained between 10 and 20° for all participants (measured during pre-test).

The starting and ending positions of the forward-lunge-long and forward-lunge-short with stride were the same, which involved standing upright with both feet together. From this position, the participant held a dumbbell weight in each hand and lunged forward with the right foot towards the ground. At right foot contact with the ground, the participant slowly flexed the right knee until maximum knee flexion was obtained (approximately 90–100° during the forward-lunge-long and 100–110° during the forward-lunge-short) as the left knee just made contact with the ground. From this position, the participant immediately pushed backward and returned to the erect standing position with the feet positioned together. A metronome was employed for all lunge variations and set at 25 bpm to help ensure the knee flexed and extended slowly at approximately 45°/s during the 90–110° knee angle range for both the lunge descent and lunge ascent.

The forward-lunge-long and forward-lung-short without a stride was performed via the same procedure as the forward-lunge-long and forward-lunge-short with a stride, except that the feet stayed stationary as the participant first flexed both knees until the lowest position of the lunge was achieved (as shown in Figure 1 and Figure 2) and then fully extended both knees to the highest position of the lunge.

### 2.3. Data Collection

Each participant arrived at the Biomechanics Laboratory 1 week after the pre-test session and changed into spandex-type shorts. The skin was then shaved, abraded, and cleaned with isopropyl alcohol wipes to reduce skin impedance, and Blue Sensor (Ambu Inc., Linthicum, MD, USA) disposable surface electrodes (type M-00-S) were applied to collect EMG data. These oval shaped electrodes (22 mm wide and 30 mm long) were placed in a bipolar configuration along the longitudinal axis of each muscle tested, with a center-to-center inter-electrode distance of approximately 3 cm. Electrode pairs were placed only on the participant’s right side, using previously described locations [15,28], for the following muscles: (a) rectus femoris; (b) vastus lateralis; (c) vastus medialis; (d) medial hamstring (semitendinosus); (e) lateral hamstring (long biceps femoris); (f) gastrocnemius (middle portion between medial and lateral bellies); (g) adductor longus; (h) gluteus maximus; and (i) gluteus medius. Reflective markers (3.8 cm diameter) were then attached and placed over the following areas: (a) right foot third metatarsal head; (b) right leg medial and lateral malleoli; (c) right knee upper edges of lateral and medial tibial plateaus; (d) posterosuperior greater trochanters of right and left femurs; and (e) right shoulder lateral acromion.

Once the reflective markers and electrodes were positioned, each participant warmed up by light walking, stretching, and practicing the exercises until they felt they were ready to be tested, and then data collection commenced. A Peak Performance 6 camera motion analysis system (Vicon-Peak Performance Technologies, Inc., Englewood, CO, USA) was employed to collect video data (60 Hz) from the reflective markers to calculate knee angle. EMG data were collected (960 Hz) from a Noraxon Myosystem unit (Noraxon USA, Inc., Scottsdale, AZ, USA). The bandwidth frequency of the EMG amplifier was 10–500 Hz and had an input impedance of 20,000 kΩ, and the common-mode rejection ratio was 130 dB. Video and EMG data were electronically synchronized and simultaneously collected as each participant used their 12 RM weight while performing 3 consecutive repetitions (trials) of the forward-lunge-short with a stride, and forward-lunge-short without a stride, forward-lunge-long with a stride, and forward-lunge-long without a stride, performed in a random order and with a 3 min rest period between exercise trials.

Subsequent to the completion of all forward lunge variations, EMG data were collected from maximum voluntary isometric contractions (MVIC) to normalize each muscle by its %MVIC. For the vastus medialis, vastus lateralis, and rectus femoris, knee extension MVIC were performed at 90° hip and knee flexions in a seated position. For the medial and lateral hamstrings, knee flexion MVICs were performed in the same seated position as for the knee extensions. For the adductor longus, hip adduction MVICs were performed in a seated position by squeezing a 21.6 cm diameter 2-ply rubber ball (Model #SP85R, Tachikara USA Inc., Sparks, NV, USA) inflated to 1.5 PSI and placed between the knees. For the gastrocnemius, unilateral standing plantar flexion MVICs were performed with the ankle positioned approximately halfway between full plantar flexion and neutral. For the gluteus medius, hip abduction MVICs were performed in the side lying position. For the gluteus maximus, hip extension MVICs were performed in the prone position, with the knee flexed 90°. For each MVIC, two 5 s trials were randomly collected, with a 2 min rest period between the trials.

### 2.4. Data Reduction

Video images for each reflective marker were tracked and digitized in three-dimensional space, and ankle, knee, and hip joint centers were mathematically determined using the external markers and appropriate equations previously described [29]. The raw position data were smoothed with a double-pass fourth-order Butterworth low-pass filter with a cut-off frequency of 6 Hz [29]. Knee joint angles were calculated by utilizing appropriate kinematic equations [29].

The raw EMG signals were full-wave rectified, smoothed using a 10 ms moving average window, and then linear enveloped during the knee range of motion for all repetitions. EMG data were subsequently normalized for each muscle as a percentage of the highest corresponding MVIC trial. The MVIC was calculated by employing the highest EMG signal over a 1 s time interval during the 5 s MVIC trials. Normalized EMG data were then averaged for the 3 repetitions (trials) between 0° and 90° during the forward lunge descent, and averaged between 90° and 0° during the forward lunge ascent.

### 2.5. Data Analysis

A repeated measures 2-way analysis of variance (ANOVA) was employed, using SPSS statistical software, version 29 (IBM SPSS, Inc, Armonk, NY, USA.). Step length (long step versus short step) and stride (with stride versus without stride) were the independent factors. The dependent variables were average EMG for each muscle between 0° and 90° for the lunge descent and average EMG for each muscle between 90° and 0° for the lunge ascent. Bonferroni *t*-tests were used for pairwise comparisons. The level of statistical significance was set at *p* < 0.01 instead of *p* < 0.05 to reduce the chance of Type I errors. Partial eta squared (ηp^2^) was employed to quantify the effect size of differences in muscle activation between different step length and stride lunge variations. Partial eta squared threshold values between 0.01 and 0.05 were considered to be a small effect, between 0.06 and 0.14 were to be considered a medium effect, and >0.14 was considered to be a large effect. 

## 3. Results

Table 1 shows mean (±SD) normalized EMG (%MVIC), *p*-values, and effect size during forward lunges with step length and stride variations for lower-extremity muscles that demonstrated no significant interactions (*p* < 0.01) between step length and stride variations. With no interactions, the mean values for the two step length variations (long and short steps) were collapsed across the two stride variations (with a stride and without a stride). Moreover, mean values for the two stride conditions were collapsed across the two step length variations. Comparing differences in step lengths (*p* < 0.01), during the lunge descent, quadriceps, hamstrings, gastrocnemius, and gluteus maximus EMG activities were significantly greater in lunges with a long step compared to lunges with a short step, while during the lunge ascent, adductor longus EMG activity was significantly greater in lunges with a long step compared to lunges with a short step. Comparing differences in stride variations (*p* < 0.01), during the lunge descent, gluteus maximus EMG activity was significantly greater in lunges without a stride compared to lunges with a stride, while during the lunge ascent, gluteus maximus and gluteus medius EMG activities were significantly greater in lunges with a stride compared to lunges without a stride.

Table 2 shows mean (± SD) normalized EMG (%MVIC) for lower-extremity muscles during forward lunges with step length and stride variations for muscles that demonstrated significant interactions (*p* < 0.01) between step length and stride variations. For the lunge descent, the following results were obtained: (1) adductor longus EMG activities were significantly greater in long-step lunges with a stride compared to long-step lunges without a stride, in long-step lunges with a stride compared to short-step lunges with a stride, and in long-step lunges without stride compared to short-step lunges without a stride; and (2) gluteus medius EMG activities were significantly greater in long-step lunges with a stride compared to short-step lunges with a stride and in long-step lunges without a stride compared to short-step lunges without a stride. For the lunge ascent, the following results were obtained: (1) quadriceps EMG activities were generally significantly greater in long- and short-step lunges with a stride compared to long- and short-step lunges without a stride, in long-step lunges with a stride compared to short-step lunges with a stride, and in short-step lunges without a stride compared to long-step lunges without a stride; (2) hamstring EMG activities were significantly greater in long-step lunges with a stride compared to long-step lunges without a stride, in long-step lunges with a stride compared to short-step lunges with a stride, and in long-step lunges without a stride compared to short-step lunges without a stride; and (3) gastrocnemius EMG activities were significantly greater in long-step lunges with a stride compared to long-step lunges without a stride, and in long-step lunges with a stride compared to short-step lunges with a stride.

## 4. Discussion

This is the first known study comparing lower-extremity muscle activity patterns as a function of step length and stride variations during a forward lunge. As hypothesized, overall lower extremity muscle EMG activity was greater in lunges with a long step compared to lunges with a short step, and greater in lunges with a stride compared to lunges without a stride.

Although 35–40 studies have qualified lower-extremity muscle activity during forward lunges [1,14,16,17,18,19,20,21,22,23,24,30,31,32,33,34,35,36,37,38,39,40,41,42,43,44,45,46,47,48,49,50,51,52,53,54], the vast majority compared muscle activity between forward lunges and a variety of other exercises, such as squats, deadlifts, step up/downs, seated knee extensions, and other exercises. The current study is the only known study that compared muscle activity between step length and stride variations during forward lunges, finding numerous step length and stride EMG differences. When collapsed across stride variations, nearly all muscle EMG (except rectus femoris) were significantly greater with a long-step compared to a short step during lunge descent, while adductor longus EMG was the only muscle significantly greater with a long step compared to a short step during lunge ascent. This implies that lower-extremity muscles are overall more active using a long-step forward lunge compared to a short-step forward lunge. When collapsed across step length variations, gluteus maximus EMG was significantly greater without a stride compared to with a stride during the lunge descent, while gluteus maximus and gluteus medius EMG were significantly greater with a stride compared to without a stride during the lunge ascent. This implies that performing the forward step by striding forward and pushing back recruits the gluteus maximum and medius to a greater degree than lunging up and down with both feet stationary.

Only approximately 20% of forward lunge lower-extremity EMG studies in the literature compared muscle activity between different lunging techniques, and like the current study, these studies also reported significant differences [1,16,17,18,19,22,24]. Three studies examined the effects of forward lunging with varying trunk positions [16,17,18], and reported mixed results. With the trunk inclined forward compared to an erect trunk, Farrokhi et al. [17] reported significantly greater gluteus maximus and biceps femoris muscle activity, while Bezerra et al. [16] reported no significant differences in these same muscles. Jonhagen et al. [18] reported greater rectus femoris, biceps femoris, and gastrocnemius muscle activity during a jumping forward lunge with the trunk inclined forward compared to a walking forward lunge with the trunk more erect. One study compared a traditional forward lunge to a suspended forward lunge, in which the rear foot was suspended in a strap above the ground [24]. These authors reported significantly greater hamstrings, gluteus maximus and medius, and adductor longus muscle activity in the traditional lunge compared to the suspended lunge, but no significant difference in rectus femoris muscle activity. One study compared forward lunging on three different surfaces: firm flat ground, unstable “STEPRIGHT” device on flat ground, and dome surface of a BOSU ball above the ground [22]. These authors reported that lunging using the “STEPRIGHT” generally produced more rectus femoris, vastus medialis oblique, and fibularis longus muscle activity compared to lunging on the remaining two surfaces. One study compared traditional (feet hip width apart) and inline (feet 50% hip width apart) forward lunging [1] and reported no significant differences between the two exercises for vastus lateralis, biceps femoris, gluteus maximus, and gluteus medius. One study compared forward lunging while holding a dumbbell on the ipsilateral side (same side as the lead lunge leg) versus holding the dumbbell on the contralateral side (opposite side to the lead lunge leg) [19] and reported significantly greater vastus lateralis and gluteus medius muscle activity when holding the dumbbell on the contralateral side compared to the ipsilateral side.

Most forward lunge EMG studies in the literature used the bodyweight lunge without sagittal plane external loading. However, in training and rehabilitation, the loaded forward lunge is also commonly employed to enhance lower-extremity muscular hypertrophy and strength. Only around 25% of the forward lunge EMG studies employed sagittal plane external loads that involved an aquabag, sandbag, elastic resistance, dumbbells, and barbells [1,14,16,19,20,21,43,48,52,53]. Most of the external loads used during the forward lunge were all different. Some studies used absolute loading for all participants, including a 4–8 kg load [53], a 10 kg load [48], a 20 kg load [21], and a 22.7 kg load [52]. Other studies used relative loading, including a 5 RM intensity (similar to 85% 1 RM) [14], a 10 RM intensity (similar to 75% 1 RM) [1,20,43], a 12 RM intensity (the current study—similar to 70% 1 RM), and 30% bodyweight intensity [16]. Because of the large variability in absolute and relative external loads employed during the forward lunge, as well as variability in how EMG data are normalized and expressed as %MVIC, it can be challenging to compare %MVIC among the forward lunge studies for various muscles. Because varying %MVIC normalization techniques can under- or over-inflate %MVIC and distort the proper interpretation of the %MVIC, it is important to look at the combined literature and establish a mean %MVIC for each muscle. The mean lower-extremity EMG values from the nine studies in the literature that employed an externally loaded forward lunge are remarkably similar to the mean lower-extremity EMG values from the current study. For example, the mean EMG for the vastus lateralis, vastus medialis, and rectus femoris from the current study were 66% MVIC, 65% MVIC, and 42% MVIC, respectively, compared to the mean EMG values for these same muscles from the literature of 69% MVIC, 66% MVIC, and 48% MVIC, respectively. Similarly, the mean EMG for the biceps femoris and semitendinosus from the current study were both 19% MVIC, compared to the mean EMG values for the biceps femoris and semitendinosus from the literature of 28% MVIC and 23% MVIC, respectively. Moreover, the mean EMG for the gluteus maximus and gastrocnemius from the current study were 38% MVIC and 22% MVIC, respectively, compared to the mean EMG values for these same muscles from the literature of 44% MVIC and 24% MVIC, respectively. The last two muscles, gluteus medius and adductor longus, were the only two that showed more substantial differences, with a mean EMG for the gluteus medius and adductor longus from the current study of 20% MVIC and 23% MVIC, respectively, compared to the mean EMG values for these same muscles from the literature of 45% MVIC and 38% MVIC, respectively. These larger differences in gluteus medius and adductor longus MVIC’s between the current study and the literature were likely due to differences in normalization techniques.

With %MVIC for lower-extremity EMG being established in the current study and in the literature during external loaded forward lunges, it is important to understand how to interpret %MVIC relative to muscle hypertrophy and strength. In a literature review on the role of resistance exercise intensity on muscle fiber adaptations, Fry [55] reported that intensities between 40 and 95% 1 RM are needed for adaptations in muscle hypertrophy and strength to occur, with the upper half of this range being most effective in maximizing muscle hypertrophy and strength. Although there is no optimal relationship between % 1 RM and %MVIC intensities, several studies have given recommendations on how to interpret %MVIC during the forward lunge and various other training and rehabilitation exercises relative to muscle intensity and strengthening [56,57,58,59]. To standardize %MVIC relative to muscle activity intensities, Escamilla et al. [58] recommended 0–20% MVIC as low-intensity muscle activity, 21–40% MVIC as moderate-intensity muscle activity, 41–60% MVIC as high-intensity muscle activity, and >60% MVIC as very high-intensity muscle activity. These authors also stated that muscle activity >60% MVIC is more effective in developing muscular strength, while muscle activity <20% MVIC is more effective in developing muscular endurance. It is generally agreed that a minimal threshold between 40 and 60% MVIC is needed to induce significant muscle hypertrophy and strength gains [56,57,58,59]. Applying these %MVIC recommendations to the EMG data from the current study and the literature would suggest that the external loaded forward lunge is effective for hypertrophy and strengthening of the quadriceps (approximately 40–70% MVIC), gluteus maximus (approximately 40–45% MVIC), and gluteus medius (approximately 20–45% MVIC). This would also suggest that the external loaded forward lunge is less effective for hypertrophy and strengthening of the hamstrings (approximately 20–30% MVIC), gastrocnemius (approximately 20–25% MVIC), and adductor longus (approximately 20–40% MVIC). These data from the external loaded forward lunge imply that from high to very high muscle activity occurs in the quadriceps, gluteus maximus, and gluteus medius, while moderate muscle activity occurs in the hamstrings, gastrocnemius, and adductor longus.

Both in the current study and studies from the literature, lower-extremity EMG was generally greater in the external loaded forward lunge [1,14,16,19,20,21,43,48,52,53] compared to the bodyweight lunge [17,18,22,23,24,30,31,32,33,34,35,36,37,38,39,40,41,42,44,45,46,47,49,50,51,52,54]. Wu et al. [21] performed the only known forward lunge EMG study in which lower-extremity EMG was compared between the bodyweight lunge and barbell, dumbbell, and weighted vest external loading lunge conditions (20 kg load used for each condition). These authors reported roughly 30–50% greater EMG in all three loading conditions compared to the bodyweight lunge for vastus medialis, rectus femoris, vastus lateralis, biceps femoris, semitendinosus, tibialis anterior, and gastrocnemius muscles, and no EMG differences among the three loading conditions for any muscle. Thus, a logical progression in rehabilitation and training is to begin with the bodyweight forward lunge when lower-intensity training is the goal, and gradually progress to greater external loads when higher-intensity training is desired, which is more conducive for muscle hypertrophy and strength training. Because overall EMG activities in the quadriceps, hamstrings, gluteus maximus, adductor longus, and gastrocnemius were greater in lunges with a long-step than lunges with a short-step, a logical lower EMG to higher EMG progression in rehabilitation and training may include starting with lunges with a short-step and progress to lunges with a long-step. Similarly, because EMG activities for the gluteus maximus and gluteus medius were greater in lunges with a stride compared to lunges without a stride during the lunge ascent, a logical lower EMG to higher EMG progression in rehabilitation and training may include starting with lunges without a stride and progress to lunges with a stride.

Even though the forward lunge is generally performed with both a descent phase (largely eccentric lower-extremity muscle actions) and an ascent phase (largely concentric lower-extremity muscle actions), only approximately 25% of studies in the literature reported lower-extremity EMG during both the descent (eccentric) and ascent (concentric) phases of the forward lunge [14,18,20,22,34,35,39,44,52]. Of these studies, Bouillon et al. [22] is the only known study that statistically compared and reported lower-extremity EMG for both descent and ascent phases while performing the forward lunge with technique variations. These authors reported significantly different rectus femoris, vastus medialis, and fibularis longus activity during both descent and ascent phases while performing the lunge on different surfaces and heights, but found no significant differences in biceps femoris, tibialis anterior, and gastrocnemius with technique variations. These results were partially similar to the current study, which reported multiple significant EMG differences between step length and stride variations during both the descent and ascent phases for the quadriceps, hamstrings, gluteus maximus and medius, gastrocnemius, and adductor longus. Muyor et al. [14] conducted the only known forward lunge EMG study that statistically compared EMG between the eccentric and concentric phases for quadriceps, hamstrings, and gluteal muscles. The mean EMG of all muscles was 31% greater during the concentric phase compared to the eccentric phase. This same pattern of greater forward lunge EMG in the concentric phase compared to the eccentric phase was also found in the current study, as well as multiple other studies in the literature [22,34,35,44,52], although EMG between eccentric and concentric phases were not statistically analyzed in these studies.

The effect sizes were large for seven out of the ten EMG values that were significantly different during the two lunge step length variations, with the remaining three EMG values exhibiting medium effect sizes and non-significant differences. The large effect sizes indicate that the significant differences found in EMG values for step length variations are meaningful. In a practical sense, during the lunge descent, this demonstrates that the long-step lunge is more effective than the short-step lunge in EMG activity in the quadriceps, hamstring, gluteus maximus, and gastrocnemius muscles. Moreover, during the lunge ascent, adductor longus EMG activity is greater in the long-step lunge than the short-step lunge. In contrast, EMG values were only significantly different with large effect sizes for three out of the ten EMG values during the two stride variations, indicating only three meaningful differences. In a practical sense, during the lunge ascent, lunging with a stride is more effective than lunging without a stride in EMG activity in the gluteus maximus and gluteus medius muscles. Moreover, during the lunge descent, gluteus maximus EMG activity is greater lunging without a stride compared to lunging with a stride.

There are some limitations in the current study. Firstly, because only healthy participants who were able to properly perform the forward lunge exercise were employed, the findings may not be generalizable to individuals with various pathologies. Secondly, the trunk position was not precisely controlled, which could have potentially influenced the muscle activation patterns during the forward lunge exercise variations, although the trunk position stayed with 10° with a range between 10-20°. Thirdly, this study only collected EMG on the lead leg during the lunge, not the rear leg, and EMG should be collected and compared on both legs in future forward lunge EMG studies.

## 5. Conclusions

Lower-extremity muscle activity is generally greater in forward lunges with a long step compared to forward lunges with a short step, and greater in forward lunges with a stride compared to forward lunges without a stride. These findings help to better characterize the EMG activity of the lower-extremity muscles during forward lunges with variations in step length and stride, and can therefore guide the clinician in a better selection of exercise prescription and progression for rehabilitation or strength training of the lower-extremity muscles. Data from the current study and the literature imply that during the external loaded forward lunge, high to very high muscle activity occurs in the quadriceps, gluteus maximus, and gluteus medius, thus enhancing muscle hypertrophy and strength in these muscles, while moderate muscle activity occurs in the hamstrings, gastrocnemius, and adductor longus. Overall muscle activity is greater during the lunge ascent (concentric) compared to the lunge descent (eccentric).

## Figures and Tables

**Table 1:** Mean (±SD) normalized EMG (%MVIC) for lower-extremity muscles during the forward lunge with step length and stride variations using a 12-repetition maximum load for muscles that demonstrated no significant interactions (*p* < 0.01) between step length and stride variations.

	Step Length Variations				Stride Variations			
Normalized EMG (%MVIC) for Lunge Descent (0–90°)	Long Step	Short Step	*p*-Value	Effect Size Partial Eta Squared η_p_^2^	With Stride	Without Stride	*p*-Value	Effect Size Partial Eta Squared η_p_^2^
Vastus Medialis	39 ± 13	33 ± 11	0.008 *	0.203	36 ± 13	36 ± 12	0.583	0.071
Vastus Lateralis	40 ± 15	31 ± 11	<0.001 *	0.440	36 ± 16	35 ± 11	0.870	0.062
Rectus Femoris	21 ± 11	20 ± 10	0.201	0.083	21 ± 11	20 ± 10	0.399	0.075
Semitendinosus	22 ± 10	13 ± 7	<0.001 *	0.693	18 ± 10	16 ± 10	0.175	0.121
Biceps Femoris	18 ± 10	8 ± 6	<0.001 *	0.746	13 ± 10	14 ± 10	0.226	0.136
Gastrocnemius	26 ± 13	15 ± 7	<0.001 *	0.760	22 ± 13	19 ± 11	0.068	0.203
Gluteus Maximus	23 ± 12	17 ± 11	<0.001 *	0.808	18 ± 11	22 ± 12	0.009 *	0.596
**Normalized EMG (%MVIC) for Lunge Ascent (90–0°)**								
Adductor Longus	32 ± 16	25 ± 15	0.002 *	0.571	32 ± 16	25 ± 15	0.078	0.561
Gluteus Maximus	53 ± 25	60 ± 29	0.457	0.130	64 ± 28	48 ± 23	<0.001 *	0.780
Gluteus Medius	26 ± 14	22 ± 10	0.140	0.867	27 ± 14	20 ± 10	<0.001 *	0.918

* Significant difference (*p* < 0.01) between step length variations and between stride variations. Note: Because there were no significant interactions between step length and stride variations, mean values for the two step length variations (long and short steps) were collapsed across the two stride variations (with and without a stride), while mean values for the two stride variations were collapsed across the two step length variations. The *p*-values shown for step length variations and stride variations represent the main effects of the ANOVA. Partial eta squared threshold values between 0.01 and 0.05 were considered to be a small effect, between 0.06 and 0.14 were considered to be a medium effect, and >0.14 were considered to be a large effect.

**Table 2:** Mean (±SD) normalized EMG (%MVIC) for lower-extremity muscles during forward lunges with step length and stride variations using a 12-repetition maximum load for muscles that demonstrated significant interactions (*p* < 0.01) between step length and stride variations.

Step Length and Stride Variations	Normalized EMG (%MVIC) for Lunge Descent (0–90°)		Normalized EMG (%MVIC) for Lunge Ascent (90–0°)					
	Adductor Longus	Gluteus Medius	Vastus Medialis	Vastus Lateralis	Rectus Femoris	Semitendinosus	Biceps Femoris	Gastrocnemius
Long Step with Stride	33 ± 15 *	21 ± 8	97 ± 29 *	99 ± 35 *	66 ± 28 *	27 ± 17 *	31 ± 17 *	29 ± 16 *
Long Step Without Stride	21 ± 11 *	21 ± 9	62 ± 19 *	63 ± 20 *	32 ± 11 *	21 ± 10 *	23 ± 11 *	20 ± 11 *
Short Step with Stride	14 ± 7	13 ± 9	84 ± 29	88 ± 30 *	65 ± 30 *	16 ± 12	16 ± 11	21 ± 11
Short Step Without Stride	10 ± 5	15 ± 11	76 ± 23	75 ± 23 *	48 ± 15 *	14 ± 7	16 ± 8	19 ± 9
Long Step with Stride	33 ± 15 *	21 ± 8 *	97 ± 29 *	99 ± 35 *	66 ± 28	27 ± 17 *	31 ± 17 *	29 ± 16 *
Short Step with Stride	14 ± 7 *	13 ± 9 *	84 ± 29 *	88 ± 30 *	65 ± 30	16 ± 12 *	16 ± 11 *	21 ± 11 *
Long Step Without Stride	21 ± 11 *	21 ± 9 *	62 ± 19 *	63 ± 20 *	32 ± 11 *	21 ± 10 *	23 ± 11 *	20 ± 11
Short Step Without Stride	10 ± 5 *	15 ± 11 *	76 ± 23 *	75 ± 23 *	48 ± 15 *	14 ± 7 *	16 ± 8 *	19 ± 9

* Significant difference (*p* < 0.01) between pairwise comparisons for each muscle.

## Data Availability

Data are contained within the article.
